# The Influence of Oxidative Stress and Natural Antioxidants on Morphometric Parameters of Red Blood Cells, the Hemoglobin Oxygen Binding Capacity, and the Activity of Antioxidant Enzymes

**DOI:** 10.1155/2019/2109269

**Published:** 2019-01-16

**Authors:** Victor V. Revin, Natalia V. Gromova, Elvira S. Revina, Anastasia Yu. Samonova, Alexander Yu. Tychkov, Svetlana S. Bochkareva, Alexander A. Moskovkin, Tatyana P. Kuzmenko

**Affiliations:** Federal State-Financed Academic Institution of Higher Education “National Research Ogarev Mordovia State University”, Saransk 430005, Russia

## Abstract

Using a wide range of different physical and chemical methods, it was found that the oxidative stress caused by addition of hydrogen peroxide to the incubation medium has a significant effect on the conformation of haematoporphyrin, influencing the oxygen-binding properties of haemoglobin in red blood cells. Morphofunctional characteristics of red blood cells change; in particular, we have observed the transformation of erythrocytes, their transition into echinocytes. In erythrocytes, in response to increased lipid peroxidation (LPO) antioxidant enzymes become active. The use of natural antioxidants (*β*-carotene and resveratrol) works towards reducting the level of oxidative processes. Resveratrol has the greatest antioxidant effect.

## 1. Introduction

The core of cells' functional activities comprises a variety of oxidative processes. The physiological state of cells depends on the intensity of their course [1]. The accumulation of an excessive amount of peroxide products triggers numerous disturbances in the activity of membrane-bound enzymes [2, 3], ion transport [4, 5], the state of receptor systems [6], the properties of the lipid bilayer of membranes, etc. [7, 8].

The main factors that regulate the rate of such processes are the formation of reactive oxygen species (ROS) [9–11], as well as the number of antioxidant compounds [12, 13] and the activity of enzymes that ensure their decay [14].

It is known that the regenerative capacity of erythrocytes of normal size is 250 times greater than their oxidative potential. However, during some pathological conditions, the antioxidant system of erythrocytes is insufficient [15]. Therefore, the topical issue is the use of natural antioxidants (AO) able to reduce the intensity of oxidative processes, and to neutralise ROS by the aid of the formation of weak compounds that are not capable of continuing the oxidation chain [16]. Natural AOs can play a significant role in the prevention of diseases associated with oxidative damage (e.g., ischaemia), and serve as an effective tool in the prevention of specific pathological processes [11, 17, 18].

Viable compounds that can effectively reduce the level of oxidative processes are *β*-carotene [19] and polyphenolic compounds, such as resveratrol [20].

And more than that, *β*-carotene is able to effectively “extinguish” peroxide radicals directly in the lipid phase of the bilayer and naturally able to have a protective effect on proteins localized in the membrane [21].

According to G. Regev-Shoshani (2003), resveratrol can interact with active forms of oxygen as a number of its glycosylated forms form water-soluble conjugates.

It is well-known that resveratrol apart from its pronounced antioxidant properties [22–24] possesses antitumour, antiviral, and vasodilating properties [25]. Resveratrol is able to activate the synthesis of antioxidant enzymes [25]. These properties make it a particularly promising natural compound for the regulation of oxidative processes in cells.

The question of the influence of *β*-carotene and resveratrol on the haemoporphyrin conformation, which, in turn, determines the oxygen-binding capacity of haemoglobin and hence, the main function of the erythrocyte, oxygen transport, was very interesting.

Given these findings, we aimed to reveal the influence of oxidative processes on the oxygen transport capacity of erythrocyte haemoglobin and the ability of antioxidants of natural origin to influence the intensity of lipid peroxidation (LPO) and morphometric characteristics of erythrocytes.

To achieve this goal, we performed the following tasks:

(i) Studying the intensity of peroxidation processes in erythrocytes in terms of accumulation of LPO products;

(ii) Studying the state of the antioxidant system of human erythrocytes;

(iii) Investigating the morphofunctional characteristics of erythrocytes and the state of bi-lipid layer of erythrocytic membranes;

(iv) Assessing the oxygen-binding capacity of erythrocytes' haemoglobin.

## 2. Materials and Methods

### 2.1. The Research Materials and Schematic of the Experiment

The research material comprised the blood of apparently healthy people (donors) obtained from the Mordovian Republican Blood Transfusion Station in Saransk. The object of this research was human peripheral blood erythrocytes. Donors were apparently healthy men (n = 53) and ranged in age from 21 to 45 years (mean age was 31 years). Their average haematological parameters: RBC was 4.6 ± 0.07 x 10^12^/l; Hb was 136.1 ± 4.34 g/l. All tests were performed in compliance with the principles of the Helsinki Declaration of the World Medical Association (WMA) [26]. The research was fulfilled following the approval of the local ethics Committee of Mordovia State University (protocol number 12 of 17 September 2014) in accordance with the principles of Good Clinical Practice.

Blood samples were divided into 2 experimental groups. The first group included a variety of check (control) samples and samples incubated with solutions of *β*-carotene (BC) and resveratrol (RSV) as antioxidants (AO). The second group included samples exposed to oxidative stress (OS) in the presence of AO.

The schematic of the experiment is presented in Table 1.

### 2.2. Method of Research

#### 2.2.1. Obtaining Packed Erythrocytes (RBCs)

Human erythrocytes were obtained by centrifuging the whole blood at 1000 g for 10 minutes. Plasma and buffy coat layers were discarded, and the precipitate was resuspended in a tenfold volume of erythrocyte incubation medium containing 10 mM of KH_2_PO_4_, 3.5 mM of KCl, 1.5 mM of MgCl_2_, 145 mM of NaCl, and 6 mM of glucose (pH = 7,4). Erythrocytes were washed three times in this medium. The resulting precipitate of red blood cells was diluted with the medium at a ratio of 1:5 (v/v). The experiment was carried out immediately after receiving the red blood cells.

#### 2.2.2. Incubation of Erythrocytes

Erythrocyte incubation was carried out in the suspension with BC or RSV for 30 minutes at 37°C and exposed to a constant inflow of an oxygen mixture. To study the effects of AO and OS three time slots, of 10, 20, and 30 minutes, were selected. The most significant changes were detected at the 30-minute exposure, so this paper presents data at this time interval. The erythrocyte suspension exposed to a similar incubation duration at 37°C served as the control sample. To create OS, H_2_O_2_ (20 *µ*M) was added to the incubation medium. We found that H_2_O_2_ in the concentration range of 1-50 *µ*M causes oxidative processes in blood cells (in particular, in erythrocytes). At the same time, if the concentration of H_2_O_2_ is less than 20 *µ*M, the pro-oxidant effect will not be sufficient to study the effects of its action; i.e., starting from 20 *µ*M, an “oxidative explosion” begins [27, 28].

It is important to note that hydrogen peroxide was added at a constant intensive mixing, which prevented a sharp local increase in its concentration in the sample.

After incubation, erythrocytes were made precipitable by centrifugation at 1000 g for 10 minutes.

To inhibit the processes caused by OS, the following AOs were used: BC and RSV. The final concentration of BC was 3.0 *µ*M [29], and that of RVS was 100 *µ*M [30].

B-carotene (≥97.0% (UV) was dissolved in dimethyl sulfoxide (DMSO) to prepare 0.3 mM solution; then it was added to red blood cells to a final concentration of 3 *µ*M. After that, the red blood cells were incubated for 30 minutes at a temperature of 37°C.

Resveratrol (≥99% (HPLC), Sigma) was dissolved in dimethyl sulfoxide (DMSO) to obtain 10 mM solution. This solution was added to the erythrocyte suspension (final concentration of 100 *µ*M).

*DMSO-Control of Erythrocytes*. When incubating erythrocyte suspension in the same amount of DMSO during 30 minutes we have not observed statistically significant changes in the morphology of erythrocytes, hemoglobin conformation, and AO status (n = 53).

#### 2.2.3. The Definition of Diene Conjugate (DC) Content in Erythrocytes by Using Z. Placer's Method (1968), as Modified by Yu. A. Vladimirov et al. (1972) and V. B. Gavrilov et al. (1983)

LPO is a chain reaction, providing expanded reproduction of free radicals, causing damage to cell membranes, modulation of apoptosis, and OS development. The specific feature of LPO is its chain character: once it begins, it sustains itself [31–34]. Due to developing oxidative damage to lipids, molecular products of LPO are formed. They are conventionally divided into primary products (hydroperoxides, diene conjugates (DC), endoperoxides) and secondary products (oxygen-containing compounds: alcohols, aldehydes, dialdehydes (the most famous MDA), ketones, lactones, epoxides) [35].

The method is based on determining the DC content in red blood cells by measuring lipid light extract absorption in the ultraviolet region of the spectrum at 232-233 nm.

#### 2.2.4. Estimation of TBARS (Thiobarbituric Acid Reactive Substances) Concentration by the H.O. Ohkawa et al. Method (1979)

Secondary products interacting with the amino groups of phospholipids and proteins that make up the membranes form polymeric fluorescent compounds, which are known as Schiff bases. In biological systems these products are usually found in rather high stationary concentrations and take part in various biochemical processes [36–38].

The method is based on the fact that, at high temperature in an acidic medium, TBARS reacts with thiobarbituric acid (TBA), forming a coloured trimethine complex with an absorption maximum at 535 nm.

We determined the TBARS concentration in erythrocyte hemolysates.

To ensure that the presence of haemoglobin does not affect the measurement results after successive dispensing of reagents and the test specimen (hemolysate) to the samples, the tubes were heated in a water bath and centrifuged, and supernatant (without protein) was taken and in it the optical density was measured.

#### 2.2.5. Estimation of Superoxide Dismutase (SOD) Activity by the C. Beauchamp, I. Fridovich Method (1971)

The essence of the method consists in determining the SOD activity needed to suppress the rate of recovery of nitro blue tetrazolium (NBT) while generating superoxide anion radical in the oxidation of xanthine by xanthine oxidase at 550 nm [39].

We determined the SOD activity in erythrocyte hemolysates. The total dilution of erythrocyte hemolysate was 1: 240.

#### 2.2.6. Estimation of Catalase Activity by the H. Aebi Method (1984)

Reduction of catalase activity in erythrocytes is observed when inflammatory processes and intoxication develop during oxidative stress. Estimation of catalase activity was carried out in erythrocyte hemolysates. The total dilution of erythrocyte hemolysate was 1: 450. The method is based on the fact that catalase destroys H_2_O_2_. The intensity of utilisation of H_2_O_2_ is measured by the sink rate of extinction at the wavelength of 240 nm, at which it has a maximum light absorption.

Enzyme activity is expressed in units of catalase activity in ml of erythrocytes per minute.

#### 2.2.7. Raman Spectroscopy (Raman Spectroscopy)

The presence of bands in the Raman spectrum during the study of conformation and Hb properties reflects its structure and functional state, allowing the estimation of the potential capabilities (capacities) of haemoglobin at the molecular level. Raman spectroscopy allows us to study the state of the iron atom and ligands bound with it, looking at the changes in the structure of the tetrapyrrolic ring of haemoporphyrin [40, 41].

To study the conformation of Hb, smears of the cell suspension on the specimen slide were prepared.

The exposure time was 1-3 seconds, the spectral data were collected three times from each erythrocyte, and simultaneously 40-50 cells were viewed from one sample using a microscope.

For visual choice of the place of registration of the Raman spectrum and to obtain the image of erythrocytes we used a Leica microscope combined with the camera.

Recording of Raman spectra was performed with the use of an inVia Basis Raman spectrometer manufactured by the Renishaw Company (UK) with a short-focus high-aperture monochromator (focal length not more than 250 mm). A laser was used to excite the Raman spectra (532 nm wavelength, 100 mW maximum radiation power, 100x lens). The data recorder was a CCD detector (1024 × 256 pixels with Peltier cooling up to -70°C), grating 1800 lines/mm.

Digitisation of spectra (correction of baseline and smoothing of Raman spectra) and mathematical processing of data were carried out using OriginPro 2015 software [41–45].

The spectrum bands of haemoglobin excited by the 532 nm laser and the bands' compliance with fluctuations and alterations of porphyrin relations were recorded. Each sample was measured three times and the resulting values were averaged.

#### 2.2.8. Statistical Processing of the Results

The statistical significance of the mean values was calculated using Student's t-test after checking the normality of the distribution of the studied parameters with Statistica 6.0. software. The value of correlation was estimated using the Pearson correlation coefficient. The significance of differences was assessed by Student's t-test. The differences were considered significant at p≤0.05. The research results are presented as the mean and standard deviation (mean ± SD).

## 3. Results

### 3.1. The Intensity of Lipid Peroxidation in Erythrocytes via Accumulation of Oxidation Products

LPO is a metabolic process represented in almost all mammalian cells, organs, and tissues [35]. H_2_O_2_ has pro-oxidant properties and is able to initiate LPO processes in blood cells, particularly in erythrocytes [46]. In our research the intensity of peroxidation processes was judged by the level of primary and secondary products of LPO and TBARS.

**Figure 1:**
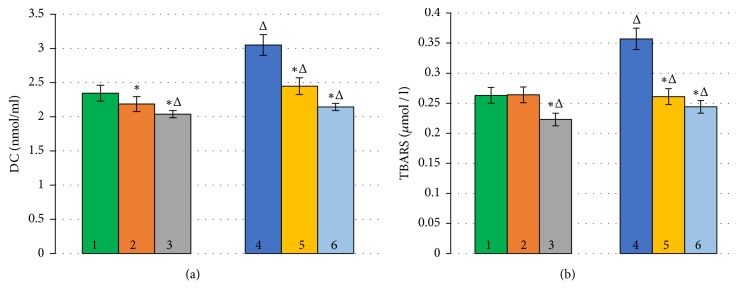
Change of ВС (a) and TBARS (b) with AOs in standard conditions and with oxidative stress (*∗*p ≤ 0.05; Δ is the change in relation to the control). (1) Control; (2) Control+*β*-carotene; (3) Control+resveratrol; (4) Н_2_О_2_; (5) Н_2_О_2_+*β*-carotene; (6) Н_2_О_2_+resveratrol.

We have shown that the DC content in the control sample of human erythrocytes was 2.347 ± 0.314 nmol/ml. Adding BC and RSV to the erythrocyte suspension decreased the number of DCs by 6.8 and 13.1% (p<0.05), respectively (Figure 1(a)).

The amount of TBARS, the final product of LPO, in the control sample of human erythrocytes was 0.263 ± 0,047 *µ*mol/l. With BC and RSV in the incubation medium during standard conditions, the amount of TBARS decreased by 5.4 and 14.4% (p<0.05), respectively (Figure 1(b)).

The findings show that, even under standard conditions peroxidation of erythrocytes occurs; consequently, AOs reveal their protective effect [47].

During OS, DC content increased 1.3 times (p<0.05) and TBARS 1.4 times (p<0.05) (Figures 1(a) and 1(b)) compared to the standard condition, which indicates an increase in the LPO processes. This may be attributed to the fact that exogenous H_2_O_2_, which is an additional source of AOs, is able to accelerate free radical formation reactions [46].

The presence of AOs in the incubation medium under these conditions facilitated the decrease in DC content by 19.8 and 29.7% (p<0.05) when adding BC and RSV, respectively, in comparison with OS conditions (Figure 1(a)). A similar effect was observed during the study of TBARS content. When BC and RSV were added, the TBARS concentration decreased by 26.9 and 31.7% (p<0.05), respectively, compared to the OS conditions (Figure 1(b)).

### 3.2. Study of Antioxidant System Values in Erythrocytes

Under physiological conditions, erythrocytes exhibit a self-sustaining activity of antioxidant defence enzymes, such as SOD, catalase, glutathione peroxidase, glutathione peroxidase, and glutathione reductase. Their coordinated actions protect erythrocyte biomacromolecules from free radicals.

The concept of the antioxidant defence system, i.e., the prevention of oxidative cell damage draws on the balanced activity of erythrocyte enzymes, is necessary to maintain redox homeostasis [15]. The work of erythrocytes' antioxidant system in our study was evaluated via changes in the activity of the main AO enzymes of erythrocytes, superoxide dismutase, and catalase.

The content of SOD in the control sample of human erythrocytes was 387.5 ± 0.4 U/ml of erythrocytes per minute, and the content of catalase was 19.74 ± 0.83 mmol/ml of erythrocytes per minute, which is consistent with the literature data, according to H. Aebi (1984) and J. Maral et al. (1977).

**Figure 2:**
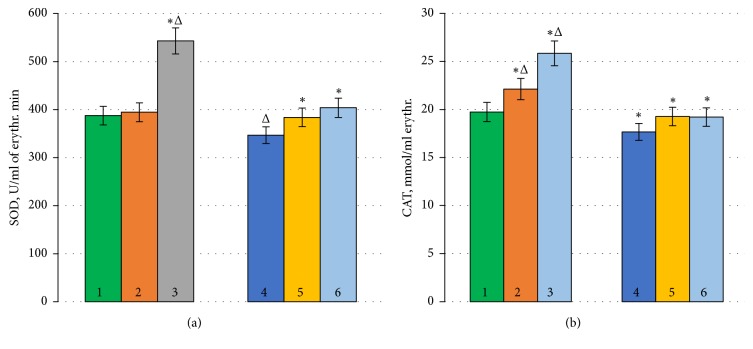
Changes in SOD and CAT activity caused by AOs under standard conditions and oxidative stress (*∗*p ≤ 0.05; Δ is the change in relation to the control). (1) Control; (2) Control+*β*-carotene; (3) Control+resveratrol; (4) Н_2_О_2_; (5) Н_2_О_2_+*β*-carotene; (6) Н_2_О_2_+resveratrol.

The addition of BC to erythrocytes during standard conditions of incubation did not cause significant changes in the activity of SOD; the values remained within the norm, but with the addition of RVS, there was a sharp increase in the activity of the enzyme by 40.2% (p<0.05) in comparison with the control sample (Figure 2(a)).

Incubation of erythrocytes with BC and RSV caused an increase in catalase activity by 12.1 and 30.9% (p<0.05), respectively (Figure 2(b)).

The findings indicate the ability of AOs under study to influence the activity of the analysed enzymes.

During OS, enzyme activity decreased by 10.5% (p<0.05) (Figure 2) in comparison with the control sample. Since enzymes have different primary structures that determine their spatial conformation and physicochemical properties, the change in activity occurs in different ways [28].

Under the same conditions, the addition of AOs to the incubation medium contributed to the increase of SOD activity almost to the control level.

Thus, the presence of BC and RSV in the sample induced increases in SOD activity by 10.7 and 16.5% (p<0.05), respectively, compared to the OS case (Figure 2(a)). In our case of the estimation of catalase activity during OS, BC and RSV caused the increase in this value relative to OS by 10.9 and 10.8% (p<0.05), respectively. In addition, the activity of the enzyme was also close to the control level (Figure 2(b)).

### 3.3. The Effects of Antioxidants and Hydrogen Peroxide on the Morphology of Erythrocytes

Deformability of erythrocytes conducive to fulfilling their basic function, oxygen transport, is directly linked to morphometric characteristics of the cell, specifically its shape [48, 49]. The morphology of erythrocytes was examined using an inVia Basis Raman spectrometer.

**Figure 3:**
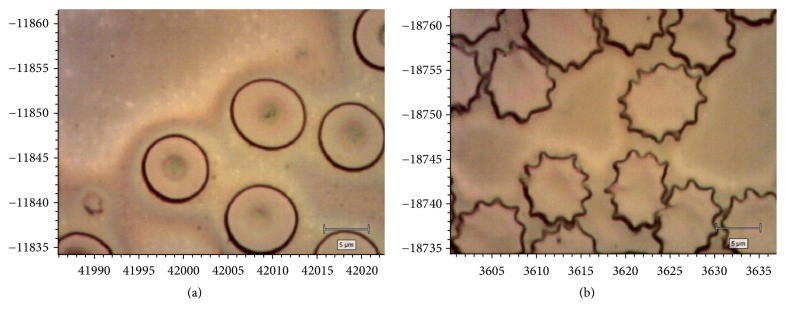
Photographs of human erythrocytes under normal conditions (a) and after incubation during oxidative stress (b).

The erythrocytes of the control group had a characteristic discoid shape, were thick at the edges, and had central concavity (Figure 3(a)). During oxidative stress caused by H_2_O_2_, most erythrocytes were represented by echinocytes of different sizes (Figure 3(b)).

It is assumed that H_2_O_2_ can modify the restructuring of the sub-membrane cytoskeleton (and, accordingly, transform the shape of erythrocytes) by forming spectrin-haemoglobin cross-links [50]. It is believed that echinocytes are the morphological sign of OS [51].

**Figure 4:**
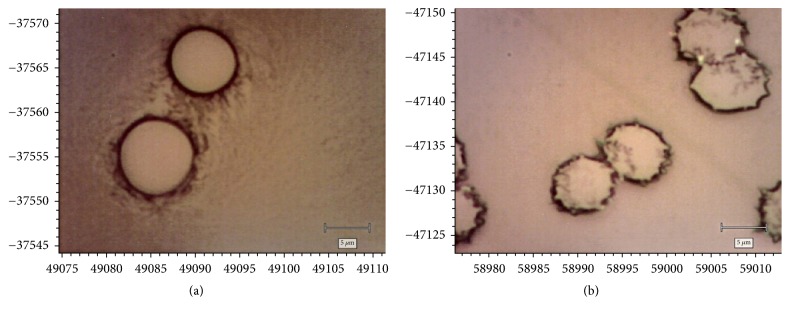
Photographs of human erythrocytes incubated with BC under normal, control conditions (a) and oxidative stress (b).

In samples incubated with BC, we observed the appearance of paraboloidal-shaped erythrocytes (Figure 4(a)).

Among the erythrocytes incubated with BC and H_2_O_2_, the formation of echinocytes was detected (Figure 4(b)).

**Figure 5:**
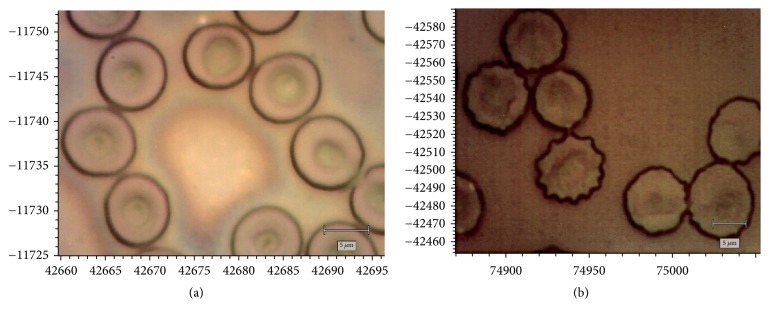
Photographs of human erythrocytes incubated with RSV under normal, control conditions (a) and oxidative stress (b).

Erythrocytes, with RSV in their incubation medium, were presented by normocytes and echinocytes in the initial stages of formation (showing the formation of a small number of spicules on the surface of cells) (Figure 5(a)).

Similar results were obtained when there was a combined effect of RSV and H_2_O_2_ (Figure 5(b)).

### 3.4. The Effects of Antioxidants and Hydrogen Peroxide on Spectral Characteristics of Haemoglobin

Changes in the morphology of erythrocytes during oxidative stress are usually accompanied by disturbances of the distribution of haemoglobin within it. Apart from that, the structure and function of haemoglobin can be disrupted, including its haematoporphyrin ring. This, in general, significantly affects the oxygen-binding properties of haemoglobin and the efficiency of O_2_ transport in the human body [45].

### 3.5. Change of Oxygen-Binding Capacity of Haemoglobin in Erythrocytes under Oxidative Stress

According to А. Kinoshita et al. (2007) one of the significant causes of disturbance in the oxygen-transport system can be a change in the conformation of haematoporphyrin and its oxygen-binding capacity in haemoglobin. Hence, we investigated the impact of OS on the conformation of Hb erythrocytes of apparently healthy persons (donors).

The position and intensity of Raman scattering (RS) bands of haemoglobin spectrum depend on variations of links in a porphyrin ring that allows you to estimate the conformation of haematoporphyrin (HP), which is directly related to hemoglobin's oxygen-binding properties [43].

To analyse the conformation of hemoglobin's haematoporphyrin (HP) we took into account specific bands of RS spectrum, which allowed us to explore the conformation of HP in deoxyhemoglobin (deoxyHb) and the ability of deoxyHb to bind ligands, as well as the HP conformation in oxyhemoglobin (HbO_2_) and the ability of HbO_2_ to reduce the oxygen.

**Figure 6:**
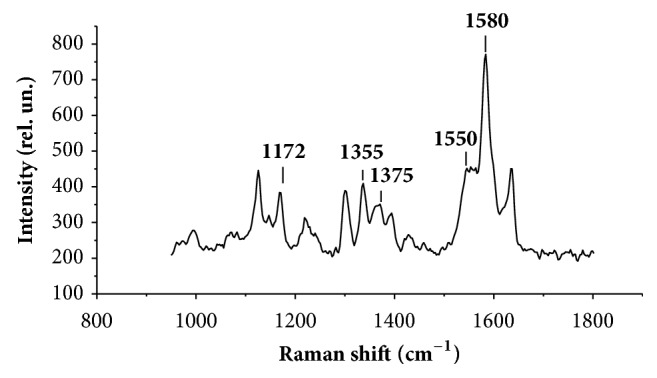
Typical Raman spectrum of an intact erythrocyte.

In this paper to analyse the conformation and Hb's О_2_–binding properties we drew on the following RS band spectra of blood (maximum positions indicated): 1172, 1355, 1375, 1550, 1580 cm^–1^ (Figure 6) [52, 53].

The use of RS-peaks correlations and not their absolute values is explained by the fact that the absolute value of spectrum intensity depends on the amount of haemoglobin, and thus amount of erythrocytes in the sample and in the laser's focus. Internal normalisation of peaks (on the intensity of other bands) ensures that the analysed parameters in different samples do not bear on the amount of hemoglobin and are determined only by the conformation of hemoglobin and the relative content of its various forms.

The character of spectra in Raman scattering (RS) of haemoglobin allows us to determine the degree of oxidation of the iron atom included, its spin state, and the presence of ligands and reflects changes in the structure of globin, leading to deformation of haemoprotein and affecting oxygen-binding properties of haemoglobin [54].

The intensity of the spectrum bands 1355 and 1375 cm^–1^ is related to symmetrical oscillations of pyrrole rings in deoxyHb molecules and haemoglobin bound with ligands, respectively [55]. As the amount of O_2_ in blood is 3-4 orders higher than the concentrations of other ligands (for example, NO or CO), the intensity of the band 1375 cm^–1^ is mainly determined by the content of oxyhaemoglobin. Therefore, the intensity ratio I_1375_/(I_1355_+I_1375_) is proportionate to the relative amount of HbO_2_ in blood [53].

The bands at 1548-1552 cm^−1^ and 1580-1588 cm^−1^ are referred to as the fluctuations and alterations of methine bridges between pyrroles in Hb molecules in which the haematoporphyrin ring either is stretched and deformed or has a more compact and undeformed conformation. In the first case, the ligand bonds are weak and held on haematoporphyrin; in the second case, the bonds are stronger [43].

The ratio of I_1355_/I_1550_ displays the relative ability of the total Hb in the sample to bind ligands [44].

The ratio of I_1375_/I_1580_ displays the relative ability of Hb to isolate ligands [56].

The ratio (I_1355_/I_1550_)/(I_1375_/I_1580_) displays the affinity of Hb to ligands, primarily to O_2_ [57].

The band at 1172 cm^−1^ appears as a result of asymmetric fluctuations and alterations of pyrrole rings in HbO_2_, and the band at 1375 cm^−1^ is symmetric. The ratio of I_1375_/I_1172_ refers to the severity of symmetric and asymmetric fluctuations and alterations of pyrrole rings, and its change is linked with conformational changes of pyrroles [41].

In intact erythrocytes, the relative amount of HbO_2_ (I_1375_/(I_1355_+I_1375_) in the control sample was 0.51 ± 0.02 rel. units, the affinity of Hb to ligands, primarily to O_2_ (I_1355_/I_1550_)/(I_1375_/I_1580_), was 0,74 ± 0,05 rel. units, the ability of Hb to bind ligands (I_1355_/I_1550_) was 0.84 ± 0.28 rel. units, the ability of Hb to isolate ligands (I_1375_/I_1580_) was 0,51 ± 0,09 rel. units, and the severity of symmetric and asymmetric fluctuations and alterations of pyrrole rings (I_1375_/I_1172_) was 0.90 ± 0.06 rel. units.

According to J. C. Merlin (1985) and C. Castiglioni et al. (2004) the Raman spectra of the most carotenoid-containing biological objects are dominated by 3 main bands with maxima at 1006 cm^−1^, 1157 cm^−1^, and 1524 cm^−1^.

The core of the carotenoid structure is a conjugated carbon chain with an average length of 20 carbon atoms containing conjugated double bonds. They perform the functions of a chromophore and determine the spectral properties of carotenoids, which have 2-3 maxima of light absorption in the range from 420 to 530 nm [58]. Following this, the spectrum of *β*-carotene can be superimposed with the Hb spectrum, and the main bands typical of the Hb spectrum can be easily identified [59].

**Figure 7:**
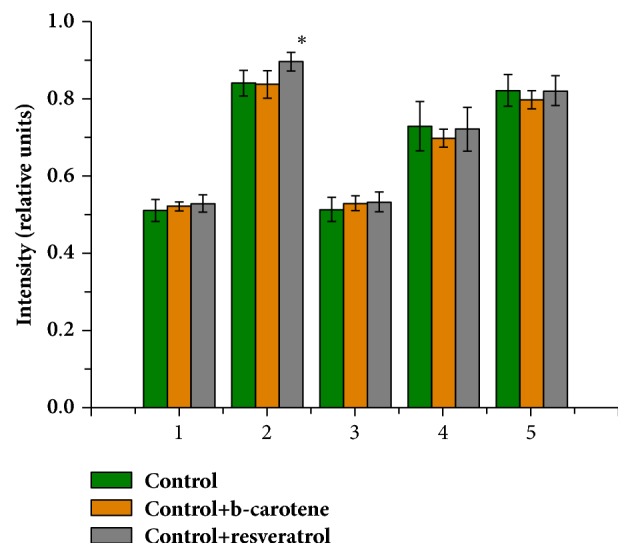
Ratio of the Raman spectrum characteristic bands of Hb in erythrocytes when exposed to AOs under standard conditions. *∗*is p≤0.05. (1) I_1375_/(I_1355_+I_1375_) – the relative amount of HbO_2_ in the blood; (2) I_1355_/I_1550_ – the ability of the total Hb in the sample to bind ligands; (3) I_1375_/I1_580_ – the ability of Hb to isolate ligands; (4) (I_1355_/I_1550_)/(I_1375_/I_1580_) – the affinity of Hb to ligands, primarily to O_2_; (5) I_1375_/I_1172_ – the severity of symmetric and asymmetric fluctuations and alterations of pyrrole rings.

When adding AOs to the incubation medium, noticeable changes in the oxygen binding capacity of Hb erythrocytes did not occur; all values remained close to the values of the control samples. With the addition of RSV to the incubation medium, there was a reliable increase in the ability of Hb to bind ligands (I_1355_/I_1550_) (Figure 7).

OS, caused by the presence of H_2_O_2_ in the incubation medium, has a significant effect on the Hb haematoporphyrin conformation, which produces a change in almost all indicators responsible for the oxygen-binding properties of Hb in erythrocytes.

**Figure 8:**
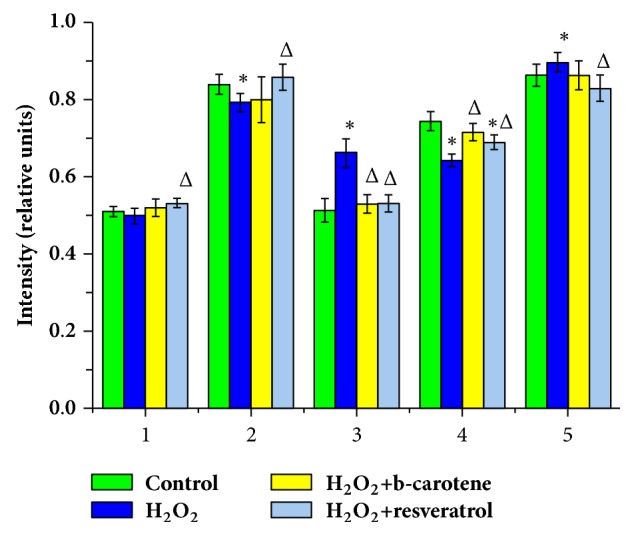
Ratio of Raman spectrum characteristic bands of Hb in erythrocytes when exposed to AOs during oxidative stress. *∗* is p≤0,05 compared to control samples. Δ is p≤0,05 compared to the OS case. (1) I_1375_/(I_1355_+I_1375_) – the relative amount of HbO_2_ in the blood; (2) I_1355_/I_1550_ – the ability of the total Hb in the sample to bind ligands; (3) I_1375_/I1_580_ – the ability of Hb to isolate ligands; (4) (I_1355_/I_1550_)/(I_1375_/I_1580_) – the affinity of Hb to ligands, primarily to O_2_; (5) I_1375_/I_1172_ – the severity of symmetric and asymmetric fluctuations and alterations of pyrrole rings.

The ability of Hb to bind ligands (I_1355_/I_1550_) during OS decreased by 6.0% (p<0.05) compared to the control value and was 0.79 ± 0.05 rel. units. The addition of RSV to the incubation medium increased this indicator by 8.9% (p<0.05) compared to the OS case. The addition of BC during OS had no impact on this indicator. At the same time, the presence of AOs in the incubation medium decreased the ability of Hb to bind ligands (I_1355_/I_1550_) almost to the normal level (Figure 8).

The ability of Hb to isolate ligands (I_1375_/I_1580_) during OS increased by 29.0% (p<0.05) compared to the control value and was 0.66 ± 0.37 rel. units. The addition of RSV and BC to the incubation medium decreased this value by 19.7% (p<0.05) compared to the OS value (Figure 8).

The affinity of Hb to ligands, primarily to O_2_ (I_1355_/I_1550_)/(I_1375_/i_1580_), decreased by 13.5% (p<0.05) compared to the control value and comprised 0.64 ± 0.17 rel. units. The addition of BC and RSV to the incubation medium increased the value of this indicator by 12.5 and 7.8% (p<0.05), respectively, compared to the OS case (Figure 8).

The values of symmetric and asymmetric fluctuations and alterations of pyrrole rings (I_1375_/I_1172_) increased by 10.3% (p<0.05) compared to the control value and was 0.90 ± 0.03 rel. units. The addition of RSV to the incubation medium decreased this indicator by 7.8% (p<0.05) compared to that in the OS case. When adding BC to the samples, there were no reliable changes in the severity of symmetric and asymmetric fluctuations and alterations of the pyrrole rings (I_1375_/I_1172_) (Figure 8).

In the sample incubated with H_2_O_2_, no significant changes were found in the relative amount of HbO_2_ (I_1375_/(I_1355_+I_1375_)) compared to control values. The addition of RSV increased the value of this indicator by 6.0% (p<0.05) compared to the OS case. When BC was added to the incubation medium, no significant changes were found, and all values remained within the norm (Figure 8).

## 4. Discussion

Oxidative stress, caused in the model experiment, by the increased concentration of hydrogen peroxide on erythrocytes triggers the development of lipid peroxidation, accompanied by a significant accumulation of primary and final products of LPO.

Oxidative processes in erythrocytes can be regulated by both enzyme systems and antioxidants belonging to different classes of bioorganic compounds.

Our findings have shown that the natural compounds *β*-carotene and resveratrol added to the erythrocyte incubation medium developed antioxidant effects. The above effect is stronger in all cases with resveratrol. The reduction of LPO products during oxidative stress in the presence of RSV may be attributed to the formation of a resonance-stabilised resveratrol's peroxide radical and a phenoxy radical. They are easily formed due to the high degree of resonance stabilisation by C-C coupling. Moreover, resveratrol can reduce the level of H_2_O_2_ because it is an electron acceptor for a number of radicals [20].

Under the same conditions, *β*-carotene is able to inhibit free radical reactions by reducing trichloromethyl peroxyl radicals transformed in the erythrocytic membrane into epoxies by the effect of oxidation. The relatively low antioxidant activity of *β*-carotene is attributed to the absence of vitamin E and selenium in the incubation medium, in which it exhibits its maximum protective effects [19, 60, 61].

The main enzyme components of antioxidant system of erythrocytes are superoxide dismutase (SOD) and CAT. The action of antioxidant enzymes is closely related to each other and is clearly balanced [13].

SOD is the first enzyme to interact with H_2_O_2_; therefore, the decrease in SOD activity during oxidative stress is probably because the enzyme consists of two metals of variable valence (Cu, Zn), and exogenous H_2_O_2_ can directly react with SOD and cause a decrease in the degree of oxidation of Cu^2+^ to Cu^1+^. This triggers “acidification” of the active site and oxidation of various substrates by an enzyme known as SOD peroxidase activity [15]. Additionally, the decrease in the antioxidant activity of this enzyme may be attributed to modification of the protein molecule of the TBARS enzyme, the increase of which was observed when we were identifying secondary products of LPO during oxidative stress.

Among the antioxidant enzymes, according to C. R. Hunt et al. (1998), CAT plays an important role in the cell's adaptive processes in response to oxidative stress. The decrease in catalase activity during oxidative stress may be attributed to the fact that, in this case, the effects of H_2_O_2_ are made through the mechanisms of cell regulation. H_2_O_2_ oxidises SH-groups of membrane receptors, and thus, there is a change in their conformations that can be the reason for the activation of receptors. The activated receptor triggers the intracellular cascade processes at the level of the adenylate cyclase system. This leads to increased cAMP levels in erythrocytes. In turn, cAMP-dependent protein kinase A, through the mechanism of de- and phosphorylation, can regulate the activity of many enzymes, including catalase and SOD.

With resveratrol and *β*-carotene, there was an increase in the antioxidant activity of SOD and catalase [62–64]. The more significant protective effect of resveratrol is explained by the fact that it can bind to metals of variable valence, which are part of the active centre of enzymes (Cu, Zn), and form complexes with them, that is, to chelate metals [64]. According F. Caruso et al. (2004) RSV is also able to inhibit the transition of the oxidation state of Cu^2+^ to Cu^1+^ in the active SOD centre and thereby reduce the formation of ROS. According to H. Chen and Al. L.Tappel (1994), the protective effect of *β*-carotene on enzymes may be related to the protection of their protein component.

The observed changes in the shape of erythrocytes exposed to the action of the above AOs can probably be caused by several reasons. In accordance with the hypothesis of the “bi-layer pair,” the shape changes (formation of planocytes, stomatocytes, spherocytes, and echinocytes) induced in erythrocytes by foreign molecules can be attributed to differential expansion of the two monolayers of the erythrocytic membrane. Planocytes and stomatocytes are formed when the molecule is embedded in the inner monolayer, whereas spherocytes and echinocytes appear when the molecule is localised in the outer layer [51]. It was revealed that resveratrol has the capacity to be embedded in the outer monolayer of the erythrocyte membrane because of its interaction with phosphatidylcholine (PC) [65]. The formation of echinocytes, apart from oxidative stress, may be attributed to this property of resveratrol.

It is known that *β*-carotene has the ability to integrate into the erythrocyte membrane [66]. According to S. T. Omaye et al. (1997), *β*-carotene has the capacity to integrate itself into the outer monolayer of the erythrocytic membrane because of its interaction with PC, similar to resveratrol. Therefore, the formation of echinocytes in the presence of *β*-carotene can also be explained not only by oxidative stress but also by the “bi-layer pair” hypothesis [51] (similar to RSV).

With increased exposure to antioxidants, erythrocytes acquire a characteristic disc-shaped form since resveratrol and *β*-carotene do not induce degenerative forms of erythrocytes (e.g., acanthocytes), which cannot be reversibly transformed into normocytes, unlike echinocytes, which preserve the integrity of the cell's cytoskeleton [51].

Using Raman spectroscopy, we have shown how some values of the oxygen binding capacity of erythrocytic haemoglobin change during oxidative stress. In general, during oxidative stress we observed a decrease in the ability of Hb to bind ligands (I_1355_/I_1550_), a decrease in the affinity of Hb to ligands, primarily to O_2_ (I_1355_/I_1550_)/(I_1375_/I_1580_), and a slight decrease in the relative amount of oxyhaemoglobin (I_1375_/(I_1355_+I_1375_)). This phenomenon can be explained by Bohr's effect and by the increased ability to isolate ligands (I_1375_/I_1580_). The reason for these changes may also be due to the increase in conformational changes in the pyrrole rings (I_1375_/I_1172_).

It is known that the smaller the conformational alterations and fluctuations, the more compact (“rigid”) the Hb structure and the lighter and faster the ligand binding, including oxygen; the more alterations and fluctuations of pyrroles, the more elongated Hb molecules become, and under these conditions, the oxygenation becomes difficult. Along with this, there are less immersion of the iron atom in the pyrrole ring, a change in the iron spin state (a transition to high-spin), and the acquisition of a less compact and deformed conformation by haematoporphyrin, which causes a weak attachment of the oxygen to haematoporphyrin and a reduced oxygen-transport function of Hb [67].

As we have shown in our experiments, oxidative stress in erythrocytes decreases their oxygen transport function, that is, the ability of Hb to bind oxygen. This process can be explained by Bohr effect. The molecular mechanism of Bohr's effect is well studied on human Hb. According to the two-state allosteric model, tetrameric Hb of vertebrates exists in equilibrium between two different conformations, which differ in their internal affinity to oxygen: tense (T-form) and relaxed (R-form).

The influence of various factors facilitates the transition of one form into another, but the dominant mechanism of transition between Hb's R- and T-forms lies in the movement of the iron atom into or away from the region of the porphyrin ring [68].

Of course, the Bohr effect is the one out of other possible mechanisms of change in the oxygen transport function of red blood cells when extracellular action of hydrogen peroxide is in place. Hydrogen peroxide can have a direct effect on the properties of the erythrocyte membrane, as well as indirect one affecting the intensity of phosphorylation of cytoskeleton proteins, phospholipase activity, protein kinases, NO-synthase, Na^+^/H^+^-exchanger, and glucose transport [69].

These changes, in turn, influence the oxygen transport function of the red blood cell, because of changes in the packing density of haemoglobin molecules in the red blood cell, the correlations between perimembrane and cytoplasmic haemoglobin fractions, changes in the hemoglobin conformation, and the ability to bind oxygen [43, 44].

Based on these findings, we assumed that hydrogen peroxide initiates several mechanisms regulating the oxygen transport function of red blood cells, including the Bohr effect.

*β*-carotene and resveratrol produce a positive effect on the values of haemoglobin oxygen binding capacity. A more significant change in values when adding resveratrol can be explained by the ability of this antioxidant to chelate metals, including iron in the Fenton reaction [63, 64].

## 5. Conclusions

Summarising our own experimental findings and those of scholarly literature, we can state that under oxidative stress there is an accumulation of LPO products, which changes not only the morphometric parameters of erythrocytes, but also the functional characteristics of hemoglobin. Parallel to this process, there is a suppression of the antioxidant enzymes activity. More pronounced effects of restoration of erythrocytes' functional characteristics take place when using resveratrol.

## Figures and Tables

**Table 1:** The schematic of experiment.

Conditions of experiment	
(incubation during 30 minutes at +37°С)	
Control	OS

Control + BC	OS + BC

Control + RSV	OS + RSV

## Data Availability

The data used to support the findings of this study are included within the article and the original data used to support the findings of this study are available from the corresponding author upon request.
